# Local immunosuppressive microenvironment underlies reduced responsiveness to imiquimod treatment in recurrent or residual cervical HSIL

**DOI:** 10.1016/j.gore.2026.102121

**Published:** 2026-05-25

**Authors:** C.L.P. Muntinga, A.J. van de Sande, M.J.P. Welters, L.F.S. Kooreman, R.L.M. Bekkers, H. van Beekhuizen, S.H. van der Burg, P.J. de Vos van Steenwijk, E.M.G. van Esch

**Affiliations:** a Department of Gynecology and Obstetrics, Catharina Ziekenhuis Eindhoven, Michelangelolaan 2, 5623 EJ Eindhoven, the Netherlands; b GROW – School for Oncology and Reproduction, Maastricht University, Universiteitssingel 40, 6229 ER Maastricht, the Netherlands; c Department of Gynecology and Obstetrics, Franciscus Gasthuis and Vlietland, Kleiweg 500, 3045 PM Rotterdam, the Netherlands; d Department of Gynecologic Oncology, Erasmus MC Cancer Center, University Medical Center, Dr. Molewaterplein 40, 3015 GD Rotterdam, the Netherlands; e Department of Medical Oncology, Oncode Institute, Leiden University Medical Centre, Albinusdreef 2, 2333 ZG Leiden, the Netherlands; f Department of Pathology, Maastricht Universitair Medisch Centrum, P. Debyelaan 25, 6229 HX Maastricht, the Netherlands; g Department of Gynecology and Obstetrics, Maastricht Universitair Medisch Centrum, P. Debyelaan 25, 6229 HX Maastricht, the Netherlands

**Keywords:** Imiquimod, Cervical high-grade squamous intraepithelial lesion, Immune microenvironment, Immunosuppression

## Abstract

**Objective:**

The topical immune modifier imiquimod is used as alternative treatment for cervical high-grade squamous intraepithelial lesions (cHSIL). While its clinical efficacy in primary cHSIL (pcHSIL) is approximately 60%, this is only 33% for recurrent or residual cHSIL (rrcHSIL) due to reasons that are not well understood. Since the immune microenvironment plays a key role in response to imiquimod in pcHSIL, we performed an in depth comparison of the pcHSIL to the rrcHSIL immune microenvironment.

**Methods:**

Transcriptome analysis was performed using the nCounter® Human PanCancer IO360™ panel on 6 pcHSIL and 6 rrcHSIL. Multispectral immunofluorescence (13 markers) was used to analyze T- and myeloid-cell composition of pcHSIL (n = 40) and rrcHSIL (n = 10). Differences in pre-treatment immune infiltrates of rrcHSIL were related to clinical response after imiquimod.

**Results:**

Transcriptomic analyses revealed lower expression of genes involved in leukocyte activation, immune cell recruitment, T cell engagement, lymphocyte and B cell infiltration in rrcHSIL compared to pcHSIL. Gene set enrichment analysis (GSEA) supported these findings, demonstrating decreased IL2-STAT 5 signaling in rrcHSIL. Immunofluorescence staining results corroborated the transcriptomic data, with rrcHSIL displaying lower intraepithelial infiltration with CD4+(Tbet + ) T cells, and (CD14 + )CD68 + CD163- M1-like macrophages, but higher numbers of CD8+(PD1 + ) T cells and CD68 + CD163 + M2-like macrophages. RrcHSIL non-responders to imiquimod exhibited an even more immunosuppressive microenvironment than complete responders, characterized by increased infiltration of CD4 + FoxP3 + regulatory T cells and (CD14 + )CD68 + CD163 + M2-like macrophages and CD14 + HLADR- monocytic myeloid derived suppressor cells.

**Conclusions:**

The limited efficacy of imiquimod in rrcHSIL may be explained by a strong immunosuppressive microenvironment.

## 1 Introduction

Cervical high-grade squamous intraepithelial lesions (cHSIL) develop as a result of persistent high-risk human papillomavirus (hrHPV) infection and have a peak incidence in reproductive aged women (Koshiol et al., 2008, Baseman and Koutsky, 2005). Standard treatment of cHSIL consists of a large loop excision of the transformation zone (LLETZ). Although LLETZ is effective in approximately 90% of pcHSIL lesions it is associated with a risk of complications and side effects. Of particular concern is the increased risk of subfertility and preterm birth in subsequent pregnancies, which increases when larger volumes of cervical tissue are excised, for example when a second or third LLETZ needs to be performed in case of recurrent or residual cervical high-grade squamous intraepithelial lesions (rrcHSIL) (Kyrgiou et al., 2017, Loopik et al., 2021). Moreover, rrcHSIL and persisting hrHPV infections can encompass many years of follow up causing stress and cancer worry in patients.

rrcHSIL occur in 5–27% of women after their first LLETZ and are associated with a ninefold increased risk of cervical cancer (Guido et al., Jun 2014, Martin-Hirsch et al., 2013, Loopik et al., Apr 2020). National and international guidelines offer no specific recommendations about the best mode of treatment and follow-up for recurrent or residual lesions (Guido et al., Jun 2014, [8]). Hence rrcHSIL are often treated with a second LLETZ, with a much lower success rate of 60.7% (van de Sande et al., 2022). Non-invasive therapies have been extensively studied, of which imiquimod is already used in clinical practice. Imiquimod induces a local immune response in the cervix by binding to toll-like receptor (TLR) 7, expressed on dendritic cells (DCs), monocytes and macrophages. Upon binding to TLR7 cytokines, including interferon (IFN)-α, IFN-γ, interleukin (IL)-6 and IL-12, are secreted. These cytokines effectuate antigen presentation by antigen presenting cells (APCs), attraction of other immune cells to the dysplastic area and stimulate a type 1 oriented T cell response, needed to target HPV infected cells and increase the hosts’ immune response (Muntinga and de Vos van Steenwijk PJ, Bekkers RLM, van Esch EMG. , 2022/3// 2022). Several studies found a complete response rate of 60% in primary cHSIL (pcHSIL) to imiquimod (Fonseca et al., 2021, Hendriks et al., 2022/4// 2022,).

One randomized controlled trial (RCT), the TOPIC-2 trial, compared imiquimod and LLETZ in women with rrcHSIL (van de Sande AJM, van Baars R, Koeneman MM, Gerestein CG, Kruse AJ, van Esch EMG, de Vos van Steenwijk PJ, Muntinga CLP, Willemsen SP, van Doorn HC, van Kemenade FJ, van Beekhuizen HJ. Topical imiquimod treatment of residual or recurrent cervical intraepithelial neoplasia lesions (TOPIC-2): A randomised controlled trial. BJOG: An International Journal of Obstetrics and Gynaecology., 2024). This single blinded, multicenter RCT randomized women between imiquimod 5% for the duration of 16 weeks, applied three times per week intravaginally or LLETZ within four weeks of rrcHSIL diagnosis. Imiquimod showed to be inferior to LLETZ in rrcHSIL, with an efficacy of only 23% in the per-protocol analysis. The study was terminated early, since futility of imiquimod treatment was assumed after the interim analysis (van de Sande AJM, van Baars R, Koeneman MM, Gerestein CG, Kruse AJ, van Esch EMG, de Vos van Steenwijk PJ, Muntinga CLP, Willemsen SP, van Doorn HC, van Kemenade FJ, van Beekhuizen HJ. Topical imiquimod treatment of residual or recurrent cervical intraepithelial neoplasia lesions (TOPIC-2): A randomised controlled trial. BJOG: An International Journal of Obstetrics and Gynaecology., 2024).

The immune microenvironment is thought to be of particular importance for response to imiquimod in pcHSIL, with a high intraepithelial infiltration of CD3 + CD8-FoxP3- T cells, CD68 + CD163- M1 like macrophages and CD11c + DCs being beneficial. In pcHSIL high intraepithelial CD4 + and Tbet + infiltration has been correlated to reduced risk of recurrence (Origoni et al., 2013). However no studies reported on rrcHSIL. Systemically, HPV-specific immune responses are seen in patients with rrcHSIL, however, these responses are ineffective and not associated with HPV16-specific secretion of Th1 or Th2 cytokines (Steenwijk and PJ, Piersma SJ, Welters MJP, Van Der Hulst JM, Fleuren G, Hellebrekers BWJ, Kenter GG, , 2008).

At present, knowledge of the immune microenvironment of rrcHSIL is limited at best. In this study the aim is to gain knowledge of the cervical microenvironment of rrcHSIL and identify key differences between pcHSIL and rrcHSIL, underlying imiquimod treatment resistance. This study performs an in-depth analysis of the cervical immune microenvironment at the RNA level, using bulk transcriptomic analyses and at the protein level using two 6-plex multispectral immunofluorescence panels.

## 2 Methods

### 2.1 RNA isolation and transcriptome analysis

To gain information about the gene expression profiles in primary and rrcHSIL, we performed transcriptomic analysis using the nCounter platform (Bruker/NanoString® Technologies). Prior to analysis, cervical tissue samples were obtained during LLETZ procedures from twelve patients visiting the colposcopy clinic of the Catharina Hospital Eindhoven after obtaining informed consent. The cohort was divided into two groups: 6 LLETZ samples from patients with a pcHSIL (cervical intraepithelial neoplasia (CIN) 3) and 6 LLETZ samples from patients with a rrcHSIL (CIN 3) who were treated with a LLETZ once before. Formalin-fixed paraffin-embedded (FFPE) tissue blocks were cut under RNAse free conditions in ten consecutive 10 μm slices. Before and after the 10 μm slices, 4 μm sections were cut for hematoxylin-eosin (HE) staining, which were annotated for dysplastic regions by a pathologist. After standard deparaffinization of the 10 μm slides, the annotated dysplastic epithelial regions were macro-dissected and RNA isolation was performed according to manufacturer’s instructions using the miRNeasy FFPE Mini Kit (QIAGEN®). RNA quantity and quality were evaluated with the use of a spectrophotometer (NanoDrop) and a bioanalyzer (Agilent). The RNA quantity was corrected using fragments ≥ 200 nucleotides in length. A corrected RNA input of 300 ng for each sample was used for the 17 h nCounter hybridization reaction at 65°C with the probes of the human PanCancer IO360 panel, according to the manufacturer’s instructions. Eight negative controls and six synthetic positive controls were included in this panel for sample input correction. Quality check was done using the nSolver 4.0 software of NanoString®. Sample-to-sample normalization was performed by making use of housekeeping genes. Benjamini–Hochberg-adjusted p-values were used to decrease the false-discovery rate.

The normalized data was subjected to gene set enrichment analysis (GSEA; Broad Institute GSEA software package, version 4.3.2) using the collections for human gene sets (Mootha et al., Jul 2003, Subramanian et al., 2005). One thousand permutations were applied. Gene sets with a p-value of < 0.05 were selected.

Gene expression data of all genes were used in the nSolver advanced analysis to estimate the infiltration of immune cells as described by Danaher et al (Danaher et al., 2017). The difference in the abundance of cell types between primary and recurrent lesions was calculated using the Mann-Whitney *U* test, a p-value of < 0.05 was used as a cut-off value for significance.

### 2.2 Multispectral immunofluorescence staining

To further identify immunological aspects of rrcHSIL and compare immune infiltrates to those of pcHSIL, multispectral immunofluorescence was performed on biopsies of patients from two other cohorts. The first cohort consisted of patients with rrcHSIL (n = 13) treated per-protocol with imiquimod in the TOPIC-2 RCT (van de Sande AJM, van Baars R, Koeneman MM, Gerestein CG, Kruse AJ, van Esch EMG, de Vos van Steenwijk PJ, Muntinga CLP, Willemsen SP, van Doorn HC, van Kemenade FJ, van Beekhuizen HJ. Topical imiquimod treatment of residual or recurrent cervical intraepithelial neoplasia lesions (TOPIC-2): A randomised controlled trial. BJOG: An International Journal of Obstetrics and Gynaecology., 2024). The second cohort consisted of patients with pcHSIL (n = 40) treated with imiquimod in the ongoing PRedICT-TOPIC trial (ClinicalTrials.gov identifier NCT05405270). In both trials, treatment efficacy was evaluated during a colposcopy 20 weeks (PRedICT-TOPIC) and 26 weeks (TOPIC-2) after the beginning of the imiquimod treatment. A LLETZ was performed in the event of persistent or progressive disease. All other histologic outcomes underwent six months of follow-up according to national guidelines ([8], van de Sande AJM, van Baars R, Koeneman MM, Gerestein CG, Kruse AJ, van Esch EMG, de Vos van Steenwijk PJ, Muntinga CLP, Willemsen SP, van Doorn HC, van Kemenade FJ, van Beekhuizen HJ. Topical imiquimod treatment of residual or recurrent cervical intraepithelial neoplasia lesions (TOPIC-2): A randomised controlled trial. BJOG: An International Journal of Obstetrics and Gynaecology., 2024). We characterized complete response (CR) by cytological reduction to Pap 1 (i.e. normal cervical cytology) at 6 months after ending imiquimod treatment and partial response (PR) as > Pap 1 (corresponding to Atypical Squamous Cells of Undetermined Significance (ASC-US) or low-grade squamous intraepithelial lesions (LSIL) in the Bethesda classification) at 6 months. Non-response (NR) was defined as persistent cHSIL at the post-treatment colposcopy or ≥ Pap 3a2 (corresponding to HSIL in the Bethesda classification) at six months.

Of the 13 patients with rrcHSIL who were treated with imiquimod in the TOPIC-2 trial, three were excluded from our analysis. One patient (NR) was excluded due to pathologic revision showing cervical low-grade squamous intraepithelial lesion (cLSIL), another (NR) was excluded due to pathologic revision showing no dysplasia, and the third patient (PR) was excluded due to a poor biopsy that was not suitable for analysis. At 6 months after the end of imiquimod treatment three patients achieved complete response, one patient had partial response and six patients were non-responders.

All FFPE slides were revised and annotated by an independent pathologist (van de Sande AJM, van Baars R, Koeneman MM, Gerestein CG, Kruse AJ, van Esch EMG, de Vos van Steenwijk PJ, Muntinga CLP, Willemsen SP, van Doorn HC, van Kemenade FJ, van Beekhuizen HJ. Topical imiquimod treatment of residual or recurrent cervical intraepithelial neoplasia lesions (TOPIC-2): A randomised controlled trial. BJOG: An International Journal of Obstetrics and Gynaecology., 2024). Two seven color multispectral immunofluorescence panels were applied, one for T cells and one for myeloid cells, as previously described by Abdulrahman et al. (Supplemental Table1A & 1B) (Abdulrahman et al., 2020). The T cell panel consisted of CD3, CD8, FoxP3, TIM3, Tbet, PD-1 and DAPI, the myeloid cell panel consisted of CD14, CD33, CD68, CD11c, CD163, HLADR and DAPI. DAPI was used as a nuclear counterstain. Tonsil slides served as positive control.

### 2.3 Quantification of immune cells in the tumor microenvironment

Whole slide images of slides stained with the multispectral immunofluorescence panels were obtained with the Vectra 3.0.5 multispectral imaging microscope (PerkinElmer) at 20x magnification. Spectral unmixing was performed with inForm 2.6 image analysis software (PerkinElmer-Akoya Biosciences). Immune cells in the tumor microenvironment were phenotyped and counted with Qupath v0.3.2 image analysis software using semi-supervised machine learning classifiers (Bankhead et al., 2017/12// 2017). First, cHSIL regions as annotated by the pathologist were selected, and trained members of the research team annotated epithelium, stroma and regions not deemed for analysis, such as blood vessels, glands and tissue and staining artifacts. The software automatically segmented DAPI + and hematoxylin + nucleated cells, depending on the staining. The software was then trained to assign a phenotype to each cell, which was visually inspected. If errors were detected, the training was further improved until all discrepancies were resolved. The exported results contained the final density for each phenotype (positive cells/mm2).

### 2.4 Statistical analysis

Statistical analysis and data visualization was performed using GraphPad Prism (Version 9, GraphPad Software, 2021) and IBM SPSS Statistics (Version 28, IBM Corp, 2021). nCounter data were analyzed using the nSolver software (version 4.0) and the advanced analysis model (version 2.0.134, NanoString, Seattle, USA).

Single genes were compared using normalized linear counts with a log2 fold change < -1 or > 1 with Mann-Whitney *U* test.

The median immune cell counts of primary and rrcHSIL, CR and NR, single genes, pathway scores and cell type profiles were compared with the non-parametric Mann-Whitney *U* test. A threshold of median cell count ≥ 10 cells/mm^2^ in at least one response group was considered biologically common and were included in the analysis (Abdulrahman et al., 2020). A two-sided p-value of < 0.05 was considered statistically significant.

## 3 Results

### 3.1 Transcriptomics reveal lower expression of genes in rrcHSIL that reflect leukocyte activation and infiltration of T cells, dendritic cells and B cells compared to pcHSIL

**Fig. 1:**
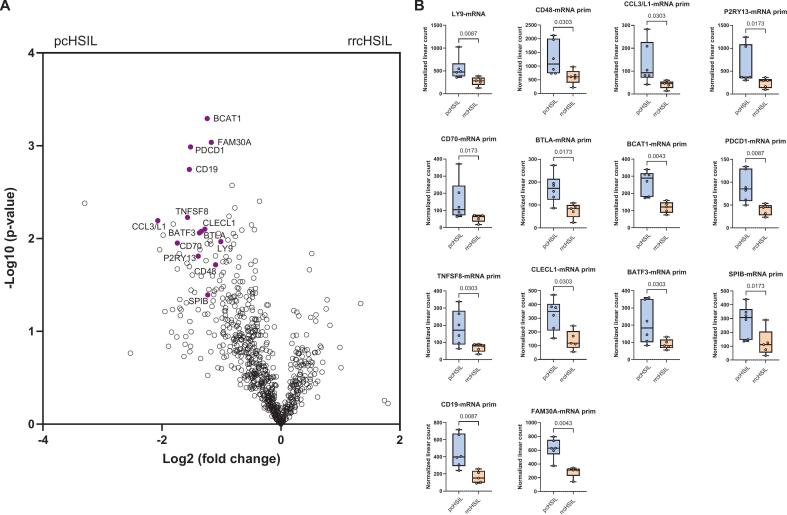
*Up- and down-regulated genes in primary cHSIL (pcHSIL) compared to recurrent or residual cHSIL (rrcHSIL).* A) Volcano plot showing higher expression of multiple genes in pcHSIL compared to rrcHSIL. Multiparametric testing did not reach significancy in differentially expressed genes between pcHSIL and rrcHSIL. B) Boxplots of upregulated single genes in pcHSIL compared to rrcHSIL, compared with Mann-Whitney U test, p-value <0.05 was considered significant.

Transcriptomic analyses were performed on RNA isolated from twelve samples, six pcHSIL and six rrcHSIL. One sample of rrcHSIL did not pass quality control. In single gene analysis, multiple genes associated with leukocyte activation and immune cell recruitment (CD48, CCL3L1, P2RY13, CD70, and BCAT1), active T cell engagement (BTLA, PDCD1, and TNFSF8), lymphocyte infiltration (LY9), presence of dendritic subsets (CLECL1, BATF3, and SPIB) and presence of B cell infiltration (CD19 and FAM30A) were expressed at lower levels in rrcHSIL compared to pcHSIL, highlighting a broader adaptive immune activation in primary lesions (Fig. 1A and Fig. 1B).

**Fig. 2:**
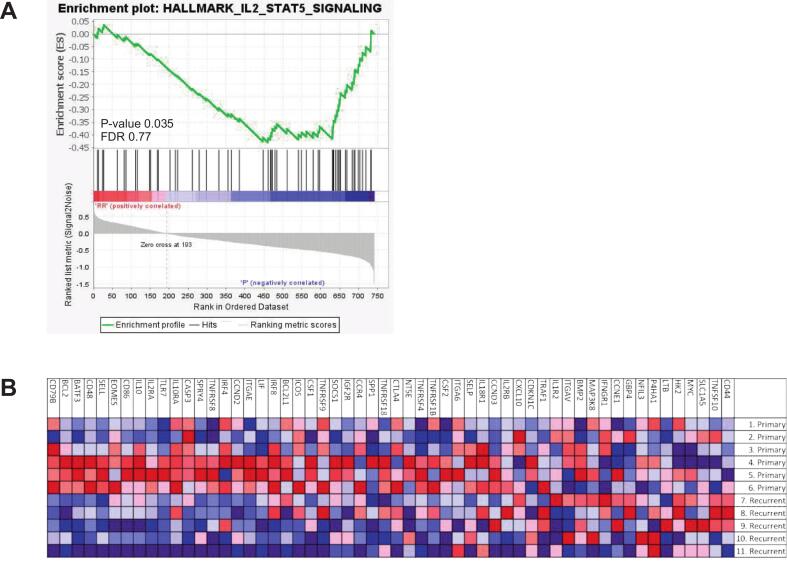
*Hallmark gene set enrichment analysis (GSEA).* GSEA analysis on primary (n=6) and recurrent or residual (n=5) cHSIL samples was performed to further identify immunological signaling events. A) Enrichment plot of IL2-STAT5 signaling, showing IL2-STAT5 signaling enrichment in primary cHSIL compared to recurrent or residual cHSIL. The black vertical lines represent the analyzed individual genes in this pathway and correspond to the genes listed in the heatmap in figure 2B. B) Heatmap of genes in the IL2-STAT5 signaling pathway per analyzed sample. Red: more enriched, blue: less enriched.

Pathway analysis of the profiled genes revealed a consistent trend toward less activation of nearly all evaluated pathways in rrcHSIL (Supplemental Fig 1, with the matrix remodeling and metastasis and antigen presentation pathway in particular (p-values 0.05 and 0.08, respectively). To further explore the underlying immunological signaling events, GSEA was conducted to provide a more detailed functional interpretation. GSEA showed significant enrichment of IL2-STAT5-signaling (p-value 0.035, false discovery rate (FDR) 0.77) in pcHSIL (Fig. 2). In contrast, in rrcHSIL GSEA showed slight enrichment of the glycolysis pathway (p-value 0.08, FDR 0.68) (Supplemental Fig 2).

**Fig. 3:**
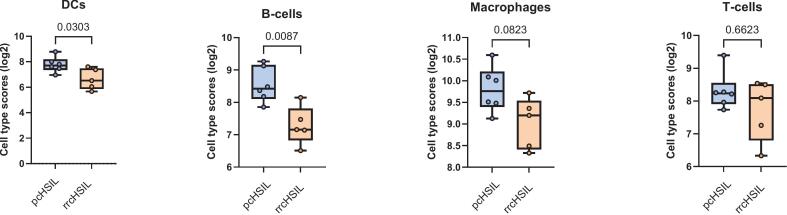
*Cell abundance differences as measured by RNA expression between primary cHSIL (pcHSIL) and recurrent or residual cHSIL (rrcHSIL).* Transcriptomic analysis was performed on pcHSIL (n=6) and rrcHSIL (n=5) to calculate the relative abundance of immune cells in both groups as described by Danaher et al [18]. In rrcHSIL a lower abundance of dendritic cells (DCs) and B-cells was observed, with a trend towards a lower infiltration of macrophages and T cells.

Finally, the gene expression results were deconvoluted to calculate the relative abundance of immune cells (Danaher et al., 2017). There was a significant lower abundance of dendritic cells and B cells and a trend towards lower abundance of macrophages in rrcHSIL compared to pcHSIL (Fig. 3), which is indicative of decreased activation of both innate and adaptive immune responses in rrcHSIL.

### 3.2 Multispectral immunofluorescent staining shows less pro-inflammatory infiltration in rrcHSIL compared to pcHSIL

**Fig. 4:**
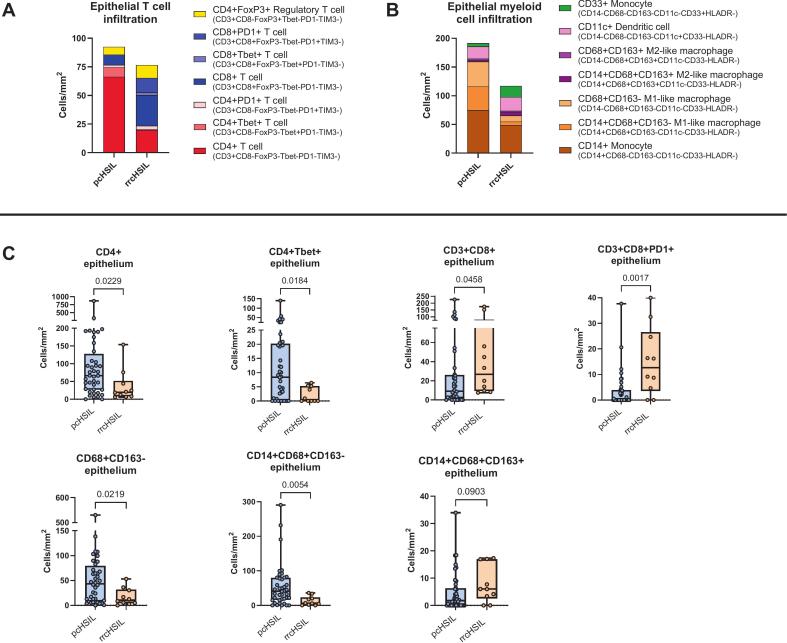
*Median intraepithelial infiltration in primary cHSIL (pcHSIL) and recurrent or residual cHSIL (rrcHSIL).* The immune infiltration of pcHSIL (n=40) and rrcHSIL (n=10) were analyzed using two 6-plex immunofluorescence panels. A) Median infiltration in T cell compartment. B) Median infiltration in myeloid cell compartment. C) Differences in T cell and myeloid cell infiltration between pcHSIL and rrcHSIL showing increased infiltration of CD4+(Tbet+) T cells and (CD14+)CD68+CD163- M1-like macrophages in pcHSIL, compared to higher infiltration of (CD14+)CD68+CD163+ M2-like macrophages and CD3+CD8+(PD1+) T cells in rrcHSIL. Groups were compared using Mann-Whitney U test.

In order to confirm our transcriptomic findings at the protein level, multispectral immunofluorescent staining was performed to study T cells and myeloid cells in the microenvironment of pcHSIL and rrcHSIL. In both groups, more than 90% of samples were hrHPV positive (p-value 1.00), the remaining samples had an unknown hrHPV status. Direct comparison revealed a lower infiltration of both the T cell and myeloid cell compartments in the epithelium of rrcHSIL (Fig. 4A and 4B). RrcHSIL were characterized by lower intraepithelial infiltration with CD4 + T cells (p-value 0.0229; Fig. 4C) and type 1 CD4 + Tbet + T cells (p-value 0.0184; Fig. 4C), while they displayed a higher intraepithelial infiltration with non-activated CD8 + Tbet-PD1-TIM3- (p-value 0.0458) and recently activated CD8 + PD1 + TIM3- T cells (p-value 0.0017; Fig. 4C). Furthermore, rrcHSIL were less infiltrated with CD14 + CD68 + CD163- and CD68 + CD163- M1-like macrophages than pcHSIL (p-values 0.0054 and 0.0219, respectively; Fig. 4C), while they showed a trend towards more intraepithelial infiltration with CD14 + CD68 + CD163 + M2-like macrophages (p-value 0.0903; Fig. 4C).

### 3.3 Non-responders to imiquimod therapy display an increased immune suppressive infiltrate

**Fig. 5:**
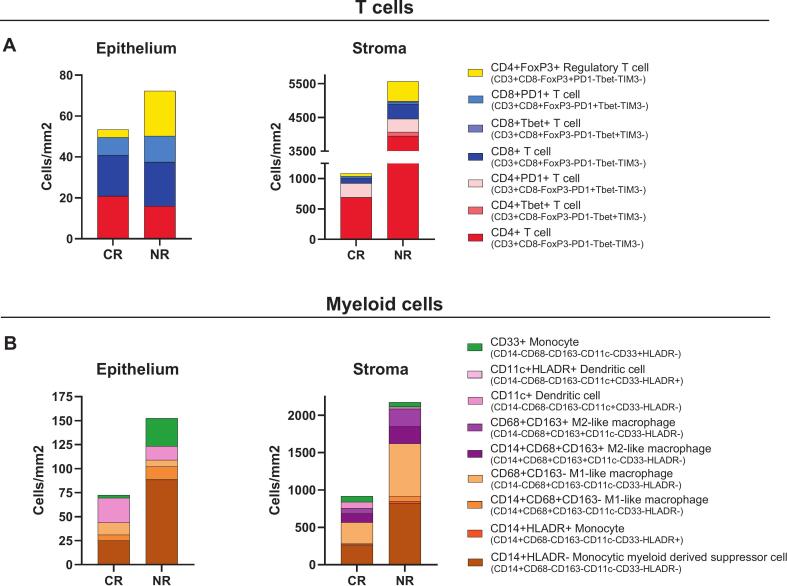
*Pre-imiquimod treatment immune infiltration in recurrent or residual cHSIL of T cells and myeloid cells stratified by response to imiquimod treatment.* The immune infiltration of recurrent or residual cHSIL (n=10) were analyzed using two 6-plex immunofluorescence panels and stratified according to response to imiquimod treatment. A) Median number of epithelial and stromal infiltrates of T cells. B) Median numbers of epithelial and stromal myeloid cells. CR: Complete Responders (n=3), NR: Non-responders (n=6). As only one partial responder was present (n=1), this case was excluded from the analysis.

To investigate immunological differences associated with treatment response, we compared the immune infiltrated in rrcHSIL between responders and non-responders to imiquimod treatment (Fig. 5, Supplemental Fig 3, Supplemental Fig 4). Non-responders in rrcHSIL exhibited a higher overall immune infiltration in both the epithelial and stromal compartments. Notably, a trend towards increased numbers of CD4 + Foxp3 + regulatory T cells (Tregs) in non-responders across both compartments was found (Fig. 5, Supplemental Fig 3). In the stroma, increased infiltration of CD4 + T helper cells and CD8 + T cells were also observed in non-responders, however only a minor subset reflects a type 1 T cell response with a CD4 + Tbet + or CD8 + Tbet + phenotype (Fig. 5, Supplemental Fig 3B). Within the myeloid cell compartment, CD14 + HLADR- monocytic myeloid derived suppressor cells (mMDSCs) were more abundant, albeit not significant, in both the epithelium and stroma of NR (Fig. 5, Supplemental Fig 4). Moreover, the epithelium of non-responders showed a trend of increased CD33 + monocytes, while the stromal compartment seems to contain higher numbers of CD68 + CD163 + and CD14 + CD68 + CD163 + M2-like macrophages (Fig. 5, Supplemental Fig 4).

## 4 DISCUSSION

To our knowledge, this is the first study to investigate in depth differences in immune microenvironment between pcHSIL and rrcHSIL using both transcriptomic RNA analysis and multispectral immunofluorescence on prospectively collected samples. In comparison to pcHSIL the rrcHSIL displayed a lower expression of genes associated with leukocyte activation, lymphocyte infiltration, dendritic cell and B cell infiltration. These transcriptomics data were supported by immunofluorescent staining results, which showed decreased overall infiltration with T cells and myeloid cells in rrcHSIL. In particular, a decrease in type 1 CD4 + Tbet + T helper cells and M1-like macrophages, coinciding with the increased infiltration of non-activated CD8 + T cells, CD8 + PD1 + T cells and immune suppressive CD68 + CD163 + M2-like macrophages.

The limited efficacy of imiquimod in rrcHSIL, with response rates of merely 30%, may be explained by the lack of pre-existent intralesional coordinated type 1 immune infiltrate, earlier shown to be associated with the response to imiquimod in both cHSIL and vulvar HSIL (van de Sande AJM, van Baars R, Koeneman MM, Gerestein CG, Kruse AJ, van Esch EMG, de Vos van Steenwijk PJ, Muntinga CLP, Willemsen SP, van Doorn HC, van Kemenade FJ, van Beekhuizen HJ. Topical imiquimod treatment of residual or recurrent cervical intraepithelial neoplasia lesions (TOPIC-2): A randomised controlled trial. BJOG: An International Journal of Obstetrics and Gynaecology., 2024, Abdulrahman et al., 2020, Abdulrahman et al., 2022/11// 2022,) and part of imiquimod’s known mechanism of action (Loopik et al., Apr 2020, Schön and Imiquimod, 2007/12// 2007,). Our data indicate that the immune microenvironment in rrcHSIL is already geared to immune suppression, and this is even more pronounced in the rrcHSIL not responding to imiquimod. These non-responding lesions are characterized by increased infiltration of Tregs, mMDSCs and CD68 + CD163 + M2-like macrophages, similar to the immune composition previously reported in non-responding pcHSIL (Abdulrahman et al., 2022/11// 2022).

While patients with rrcHSIL appear to be able to locally mount an immune response, this is insufficient to spontaneously resolve the lesion. Potentially because HPV16 specific T cells were shown to lack the production of pro-inflammatory cytokines in these patients (Steenwijk and PJ, Piersma SJ, Welters MJP, Van Der Hulst JM, Fleuren G, Hellebrekers BWJ, Kenter GG, , 2008). This notion is sustained by our observation of decreased IL2-STAT5 signaling in rrcHSIL. This pathway is known to play a pivotal role in promoting type 1 T cell differentiation and effector cytokine production, including IFN-γ (Jones et al., 2020). In addition, we observed low numbers of Tbet-expressing T cells in rrcHSIL.

The use of imiquimod as a method to heat up the local immune microenvironment (Galon and Bruni, 2019, Gerard et al., 2021) in rrcHSIL is not capable of overcoming the immune suppressive environment, comprising of regulatory T cells, M2-like macrophages and the expression of the checkpoint PD-1. A potential approach is the use of neo-adjuvant immune checkpoint blockade, such as anti-CTLA-4 to reduce the numbers of Tregs (Jiang et al., 2025) and as such potentially also CD163 + M2-like macrophages (Sasaki et al., Jan 2024) in combination with PD-1/PD-L1 inhibitors to restore CD8 + T cell function, prior to, or even replacing imiquimod treatment. PD-1/PD-L1 inhibitors (e.g. pembrolizumab) are already used in cervical cancer (Colombo et al., 2021, Lorusso et al., 2024). While side effects should not be underestimated, their use may be justifiable in patients with rrcHSIL, when conventional therapies show limited efficacy. A less invasive and non-immunological approach that may warrant further investigation is the restoration of the vaginal microbiome (VMB), for example through probiotics or treatment of bacterial vaginosis to a lactobacillus-dominant VMB, which have been associated with a pro-inflammatory immune profile and reduced HPV persistence (Schellekens et al., 2025, Yang et al., 2024).

This study has several limitations. Multispectral immunofluorescence and RNA analysis were conducted on different sets of samples, which unfortunately prohibited direct paired comparisons. Nevertheless, the consistent pattern observed across both analyses reinforces the validity of the findings. Additionally, the relatively small sample size limits the statistical power of the findings. However, despite these constraints, the study provides valuable insights into the local tumor microenvironment of rrcHSIL and highlights the differences with pcHSIL possibly underlying the reduced response to both treatment by LLETZ and imiquimod in rrcHSIL. The FDR of GSEA exceeded 25%, likely due to the sample size and the panel size consisting of 750 genes, which covers only a subset of all expressed genes included in GSEA analysis, and thus should be interpreted with caution (Mootha et al., Jul 2003, Subramanian et al., 2005). Nonetheless, the observed trends lay the groundwork for future studies with larger cohorts through longitudinal studies, as planned for in the PRedICT-TOPIC trial were a larger cohort of rrcHSIL is being recruited (ClinicalTrials.gov ID NCT05405270).

## 5 Conclusions

Our findings indicate that pcHSIL and rrcHSIL differ in intralesional immune activation and infiltration profiles, with rrcHSIL displaying an immune microenvironment that is geared to immune suppression, a phenotype that is even more pronounced in non-responders to imiquimod. Alternative treatment strategies to overcome the immunosuppressive microenvironment in rrcHSIL are warranted to treat rrcHSIL and prevent progression to cervical cancer.

## CRediT authorship contribution statement

**C.L.P. Muntinga:** . **A.J. van de Sande:** Writing – review & editing, Data curation, Conceptualization. **M.J.P. Welters:** Writing – review & editing, Visualization, Resources, Formal analysis, Data curation. **L.F.S. Kooreman:** Writing – review & editing, Resources. **R.L.M. Bekkers:** Writing – review & editing, Supervision, Conceptualization. **H. van Beekhuizen:** Writing – review & editing, Conceptualization. **S.H. van der Burg:** Writing – review & editing, Supervision. **P.J. de Vos van Steenwijk:** Writing – review & editing, Supervision, Conceptualization. **E.M.G. van Esch:** Writing – review & editing, Supervision, Resources, Methodology, Conceptualization.

## 6 Ethics approval and consent to participate

The study was conducted in accordance with the Declaration of Helsinki Consent for publication. Ethical approval was obtained from the Medical Ethics Committee of the Erasmus Medical Centre for the TOPIC-2 trial (NL53792.078.15/METC‐2015‐389) and from the Medical Research Ethics Committees United for the PRedICT-TOPIC trial (NL79879.100.22/R22.013). Participants participating in the TOPIC-2 trial and PRedICT-TOPIC trial gave informed consent to participate in the study before taking part.

## 7 Funding

CM and EvE received a research grant from Catharina Onderzoeksfonds. SvdB received base funding from Oncode Institute.

## Declaration of competing interest

The authors declare that they have no known competing financial interests or personal relationships that could have appeared to influence the work reported in this paper.
