# Supplementary material for: Local immunosuppressive microenvironment underlies reduced responsiveness to imiquimod treatment in recurrent or residual cervical HSIL
Source: Gynecol Oncol Rep. 2026 May 25;65:102121. doi: 10.1016/j.gore.2026.102121 (PMC13241649; doi:10.1016/j.gore.2026.102121)
Supplement: Supplementary Data 1 — Supplementary tables and figures. [file mmc1.docx]

**Supplemental material**

*Supplemental Table 1. Panel designs.*

A) Multispectral immunofluorescence T cell panel

| **Marker** | **Primary antibody clone and producer** | **Species and isotype** | **Dilution** | **Detection method** |
| --- | --- | --- | --- | --- |
| **PD-1** | D4W2J - CST | rabbit IgG | 1:1000 | Indirect with OPAL 570 |
| **Tbet** | D6N8B XP - CST | rabbit IgG | 1:2000 | Indirect with OPAL 520 |
| **FOXP3** | 236A/E7 - Invitrogen | mouse IgG1 | 1:25 | Indirect with CF Goat-a-Mouse IgG1 633 |
| **CD8** | 4B11 - Novocastra | mouse IgG2b | 1:50 | Indirect with Alexa fluor Goat-a-Mouse IgG2b 647 |
| **TIM3** | D5D5R XP - CST | rabbit IgG | 1:25 | Indirect with Alexa fluor Goat-a-Rabbit IgG 680 |
| **CD3** | D7A6E - CST | rabbit IgG | 1:50 | Directly labelled with Alexa fluor 594 |

B) Multispectral immunofluorescence myeloid cell panel

| **Marker** | **Primary antibody clone and producer** | **Species and isotype** | **Dilution** | **Detection method** |
| --- | --- | --- | --- | --- |
| **CD14** | D7A2T - CST | rabbit IgG | 1:25 | Indirect with CF Donkey-a-Rabbit IgG 633 |
| **CD33** | PWS44 - Novocastra | mouse IgG2b | 1:25 | Indirect with Alexa Goat-a-Mouse IgG2b 647 |
| **CD163** | 10D6 - Invitrogen | mouse IgG1 | 1:10 | Indirect with CF Goat-a-Mouse IgG1 680 |
| **HLADR** | TAL1B5 – Abcam | mouse IgG1 | 1:50 | Directly labelled with Alexa fluor 488 |
| **CD11c** | EP1347Y - Abcam | rabbit IgG | 1:100 | Directly labelled with Alexa fluor 546 |
| **CD68** | D4B9C XP - CST | rabbit IgG | 1:50 | Directly labelled with Alexa fluor 594 |


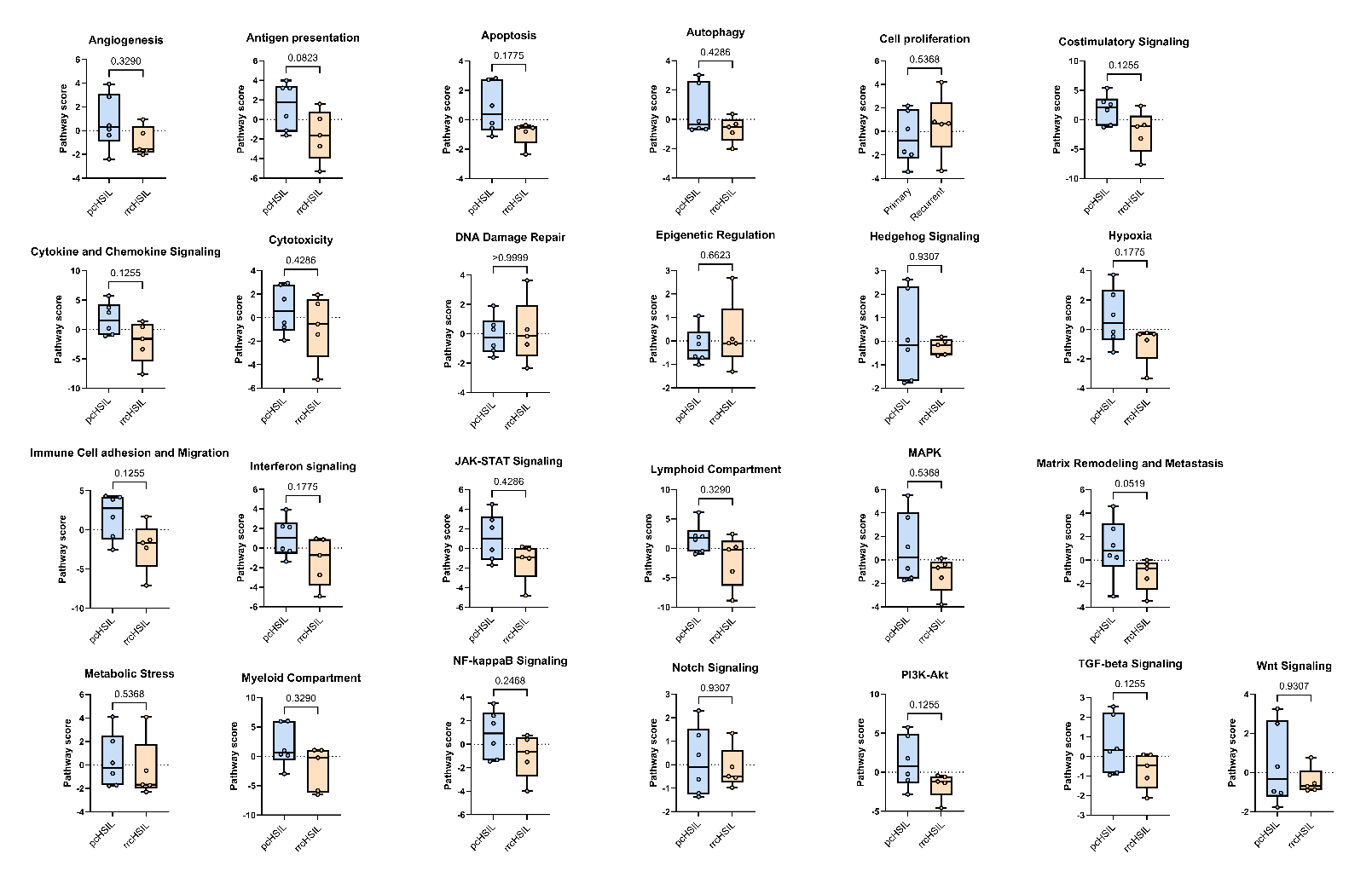


*Supplemental Figure 1. Comparison of pathway scores between primary and recurrent or residual cHSIL.* Transcriptomic analysis was performed on 6 primary cHSIL (pcHSIL) and 5 recurrent or residual cHSIL (rrcHSIL). Pathway analysis revealed a consistent trend towards less activation in rrcHSIL compared to pcHSIL, especially in the antigen presentation and matrix remodeling and metastasis pathways.

*
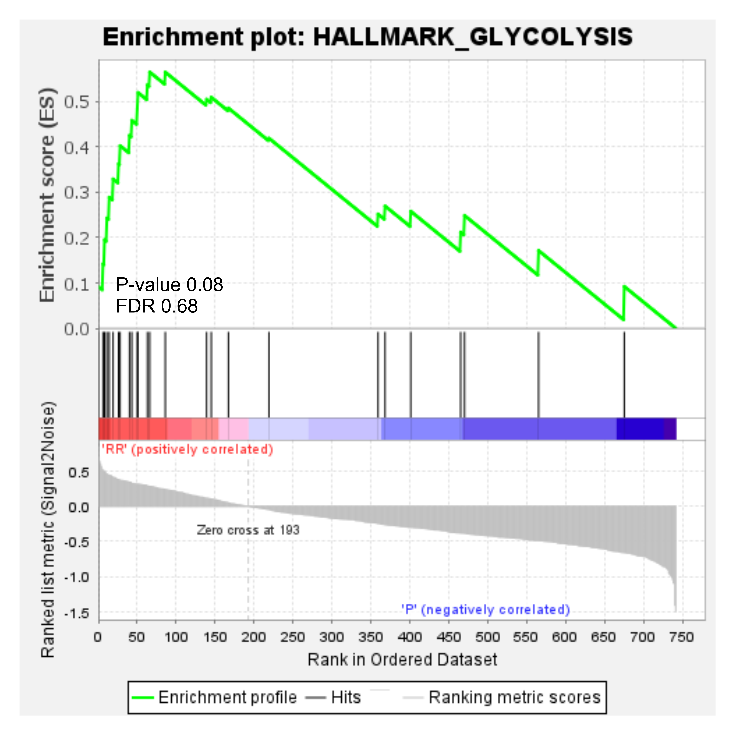
*

*Supplemental Figure 2. Hallmark gene set enrichment analysis (GSEA) plot of glycolysis pathway.* GSEA analysis on primary (n=6) and recurrent or residual (n=5) cHSIL samples was performed to further identify immunological signaling events. The enrichment plot shows increased enrichment of glycolysis in recurrent or residual cHSIL.

*
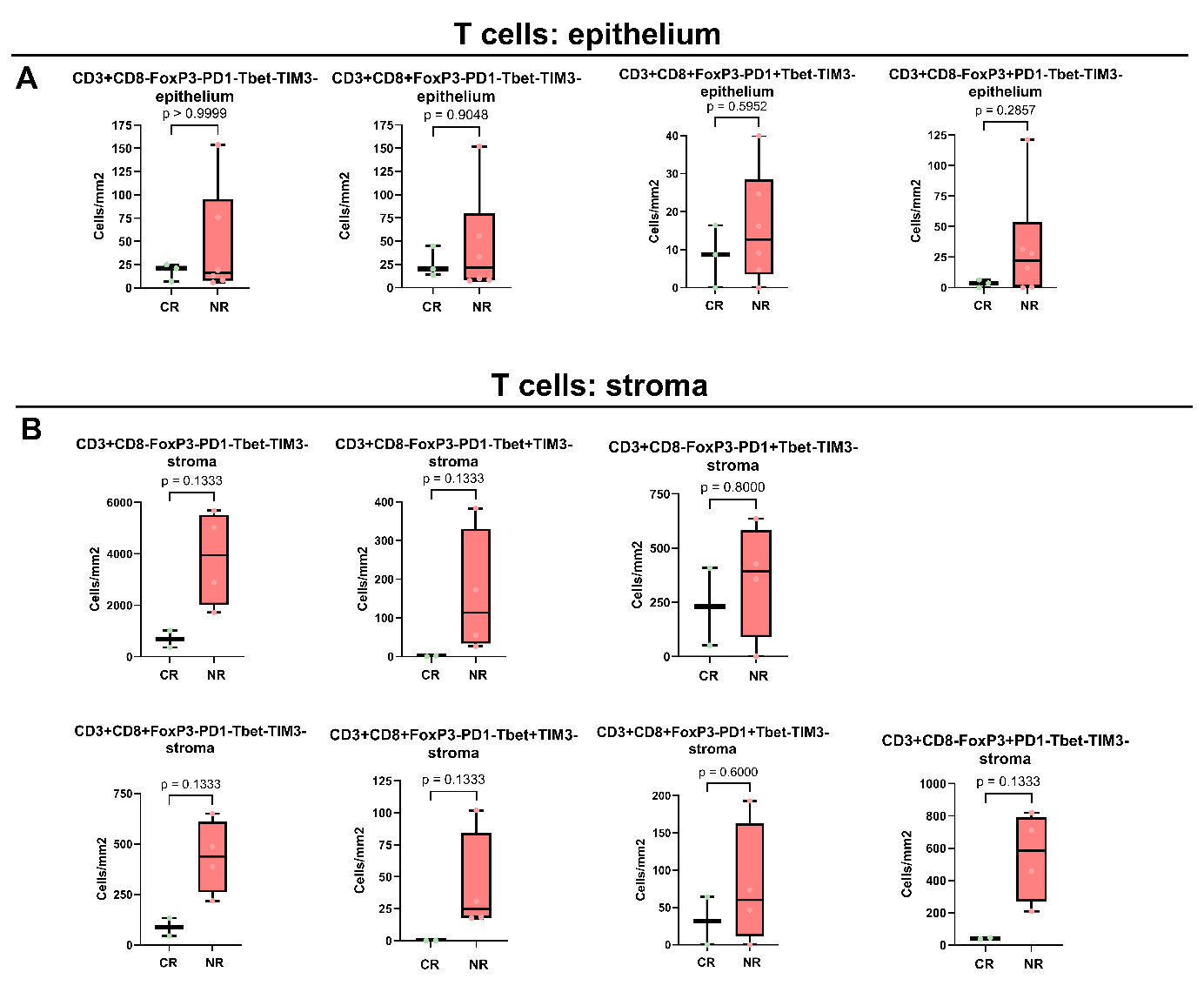
*

*Supplemental Figure 3. Pre-existing T cell infiltration in epithelium and stroma of recurrent or residual cHSIL, compared between response groups.* The immune infiltration of recurrent or residual cHSIL (n=10) were analyzed using two 6-plex immunofluorescence panels and stratified according to response to imiquimod treatment. Pre-imiquimod differences in specific immune cell phenotypes in A) epithelium and B) stroma of Complete Responders (CR) and Non-Responders (NR). Response groups were compared using Mann-Whitney U test.

*
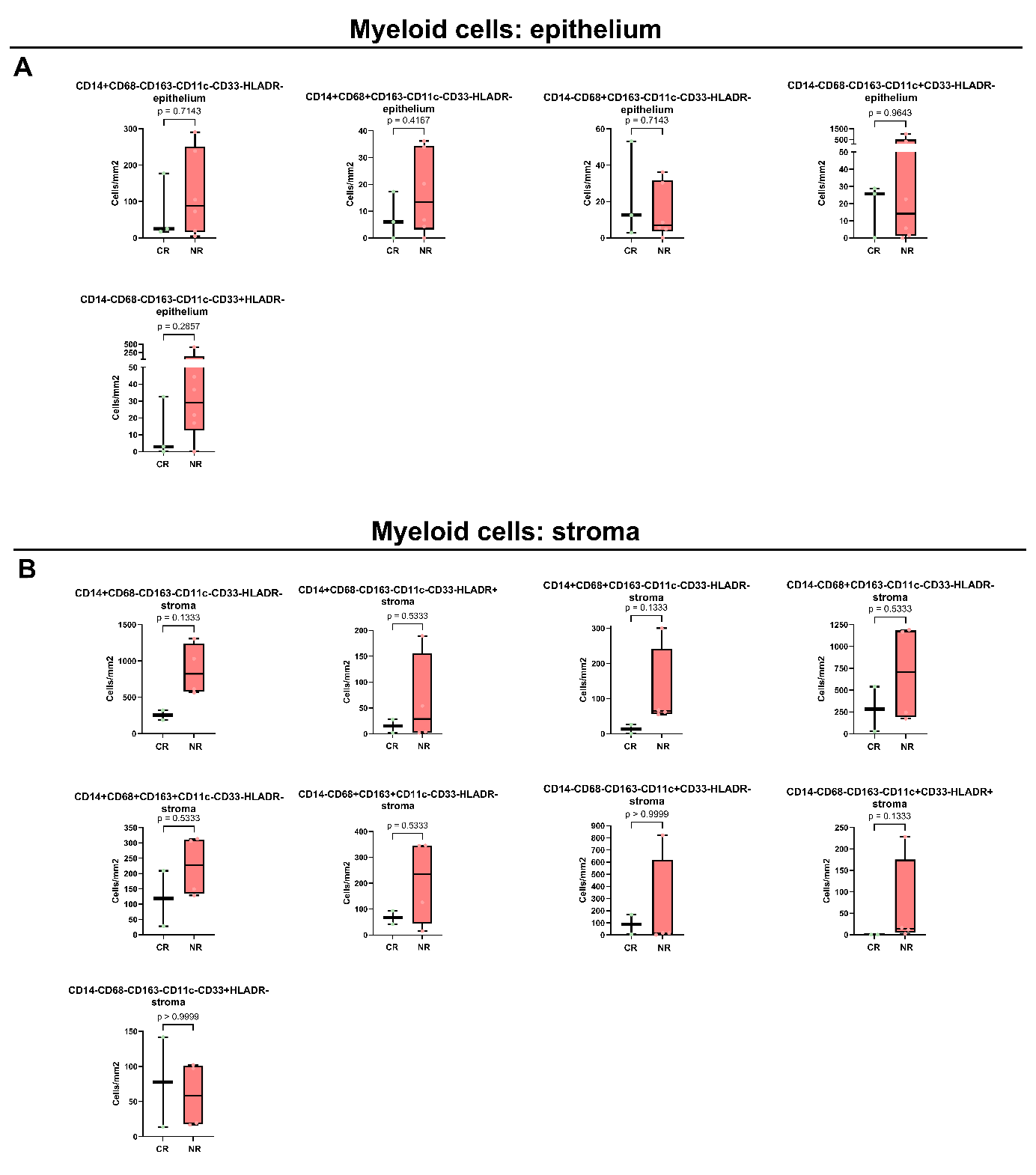
*

*Supplemental Figure 4. Pre-existing myeloid cell infiltration in epithelium and stroma of recurrent or residual cHSIL, compared between response groups.* The immune infiltration of recurrent or residual cHSIL (n=10) were analyzed using two 6-plex immunofluorescence panels and stratified according to response to imiquimod treatment. Pre-imiquimod differences in specific immune cell phenotypes in A) epithelium and B) stroma of Complete Responders (CR) and Non-Responders (NR). Response groups were compared using Mann-Whitney U test.
